# Sodium-Glucose Cotransporter 2 Inhibitor Use in Adults Undergoing Peritoneal Dialysis: A Propensity-Matched Real-World Data Analysis

**DOI:** 10.3390/jcm14248815

**Published:** 2025-12-12

**Authors:** Eric Amelunxen, Hauke S. Wülfrath, Friedrich A. von Samson-Himmelstjerna, Christoph B. Niehus, Benedikt Kolbrink, Kevin Schulte, Roland Schmitt, Laura Katharina Sievers

**Affiliations:** Department of Nephrology and Hypertension, University Hospital Schleswig-Holstein, Campus Kiel, 24105 Kiel, Germany; eric.amelunxen2@uksh.de (E.A.);

**Keywords:** sodium-glucose cotransporter 2 inhibitor, kidney failure, peritoneal dialysis

## Abstract

**Background**: Sodium-glucose cotransporter 2 inhibitors (SGLT2is) have become key therapeutic agents based on their protective cardiovascular and renal effects. However, their safety and efficacy in patients with kidney failure, especially undergoing peritoneal dialysis (PD), who are a population at very high cardiovascular risk, remain largely unexplored. **Methods**: We conducted a retrospective global cohort study using the electronic health records of 19,871 adult peritoneal dialysis patients from the Global Collaborative Network TriNetX database. Of these, n = 412 patients used SGLT2is within 3 months after PD initiation. After propensity score matching, n = 367 patients per cohort were evaluated for cardiovascular risk, mortality and adverse events related to SGLT2is-treatment. **Results**: The mean age of PD patients with SGLT2is use was 58.7 years and common comorbidities were heart failure (74.0%) and type 2 diabetes (62.1%). Comedication included beta blocking agents (75.5%), diuretics (74.0%), statins (64.6%), insulins (60.2%) and renin- angiotensin blockade (56.3%) in the majority of patients. After propensity score matching, SGLT2is use showed a trend towards reduced all-cause mortality or major adverse cardiovascular events but no significant risk reduction. Further, incidence of hemodialysis was not lowered by SGLT2is use. Known adverse events of SGLT2is use such as ketoacidosis, genitourinary infections, dehydration or peritonitis were not increased among users. **Conclusions**: In this cohort of PD patients with high cardiovascular and metabolic risk factors, SGLT2is use was safe with regard to unchanged adverse events, while effects on mortality, cardiovascular outcomes, and technique failure were neutral.

## 1. Introduction

Sodium-glucose cotransporter 2 inhibitors (SGLT2is) have evolved from glucose-lowering agents to a cornerstone of cardiovascular and chronic kidney disease (CKD) care [1]. Beyond their initial use in patients with type 2 diabetes (T2DM), SGLT2is have demonstrated significant benefits including lower risk of hospitalizations for heart failure (HF), preservation of kidney function, and improved mortality outcomes in individuals without T2DM [2,3,4]. These effects are believed to arise from hemodynamic, anti-inflammatory, and metabolic mechanisms independent of glycemic control [5,6], positioning SGLT2is as disease-modifying agents across a wide range of patient populations.

One population notably absent from this growing body of evidence are patients with kidney failure (KF). Patients with KF require kidney replacement therapy (KRT) to maintain solute clearance and fluid balance. To compensate for lack of kidney function, patients receive hemodialysis (HD) using extracorporeal dialyzers or peritoneal dialysis (PD) utilizing the peritoneal membrane and regular fluid exchanges. Once PD is established, long-term technique survival is challenging due to infectious complications, ultrafiltration failure and dialysis inadequacy, leading to transition to hemodialysis. This change is associated with increased mortality [7]. At the same time, this population typically carries a very high risk for cardiovascular disease and overall mortality and, thus, might particularly benefit from SGLT2i therapy [8,9]. However, the role of SGLT2is in KF has been questioned. Safety concerns including urinary tract infections and ketoacidosis further limit SGLT2i adoption in vulnerable populations like dialysis patients [10,11]. Emerging evidence, although observational, supports the potential use of SGLT2is in patients on KRT, while data on PD is limited and mostly derived from small case series or animal models [12,13]. To address these circumstances, we aimed to characterize real-world use of SGLT2is in PD patients and explore potential signals of benefit or harm. Therefore, we conducted a retrospective propensity score-matched (PSM) analysis using the global TriNetX research network. This study addresses the following key questions:

(1) Are SGLT2is associated with changes in all-cause mortality or major adverse cardiac events (MACE) in patients undergoing PD?
(2) Is SGLT2i therapy safe in patients undergoing PD?

## 2. Materials and Methods

### 2.1. Study Design

This retrospective cohort study utilized the TriNetX Global Collaborative Network to assess the use of SGLT2is in patients initiating PD [14]. Data were retrieved between 22 September and 10 October 2025 from approximately 184 million patients across 157 healthcare organizations. The study followed the STROBE reporting guidelines. Data used in this study is de-identified prior to researcher access; therefore, no individual patient consent or separate IRB approval was required.

### 2.2. Study Population

**Figure 1:**
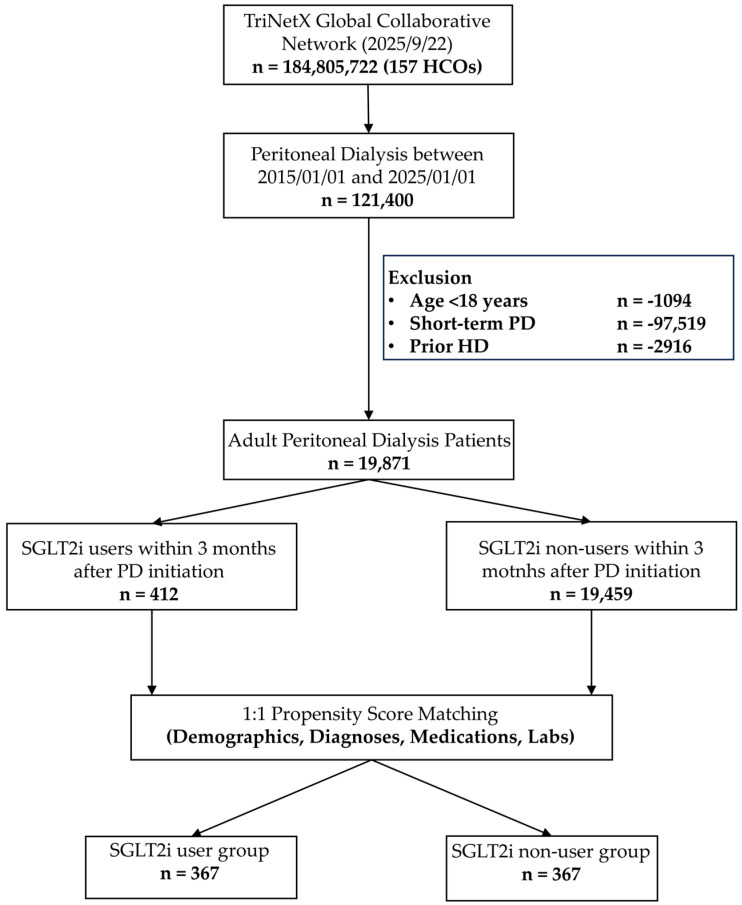
Flow chart of cohort selection process. HCO, Health care organization, PD, peritoneal dialysis, HD, hemodialysis, SGLT2is, Sodium-glucose cotransporter 2 inhibitor.

This study included adults aged 18 to 90 years between 1 January 2015 and 1 January 2025, if their electronic health records (EHR) contained at least two PD instances, identified through systematized nomenclature of medicine (SNOMED) procedure codes, PD-catheter insertion, or PD fluid prescription, separated by a minimum of three months. Patients with prior hemodialysis were excluded. The index date was defined as the first PD documentation. Cohorts were defined by SGLT2is initiation within three months after PD onset (users vs. non-users). The full query code is provided in Table S1, and the cohort selection algorithm in Figure 1.

### 2.3. Outcomes

The primary outcome of this study was all-cause mortality. The secondary outcome was MACE, defined as a composite of myocardial infarction, stroke, and cardiovascular death. Additional endpoints included ketoacidosis, osteoporotic fracture, thrombosis, amputation below the knee, urinary tract infection, dehydration, hypoglycemia, hypervolemia, peritonitis, and PD-catheter complications. Furthermore, we analyzed as a surrogate for PD technique failure the transition to HD defined as a first instance of HD procedure. Follow-up ranged from one day to five years after the index date. Detailed ICD-10 and procedural codes for outcomes are listed in Table S2. Patients with outcome event prior to the index date were excluded.

For sensitivity analyses, outcomes were evaluated at 1-, 3-, and 5-year follow-up intervals and for long-term SGLT2i users (2 prescriptions ≥ 3 months apart after PD initiation). Additionally, we performed landmark analysis involving different follow-up initiation times of 14, 30, 60, 90 and 180 days [15,16]. To further evaluate the clinical relevance of SGLT2i continuation at the initiation of PD, we conducted an additional analysis defining a cohort of patients with documented SGLT2i use prior to PD initiation, identified by SGLT2i medication code preceding the first PD record. To assess the ability of our study to detect clinically relevant differences in all-cause mortality, we performed a sensitivity analysis based on the number of observed events using the Schoenfeld approximation. The detailed numerical results of this analysis are presented in the Section 3.

### 2.4. Covariates and Statistical Analysis

Statistical analysis was conducted using the analytical tools of the TriNetX platform [14]. Cohort baseline characteristics before and after PSM were summarized as means with standard deviations (SD) and as counts with corresponding percentages [17,18]. Baseline characteristics were derived from the 6-month period before the index event. For PSM, 40 covariates including cardiovascular and metabolic diagnoses, medications and laboratory parameters were used for the built-in TriNetX PSM tool. In detail, propensity scores are calculated for each patient by logistic regression, following a greedy nearest neighbor matching algorithm with a caliper of 0.1 pooled standard deviation. Therefore, patients with standard deviations > 0.1 are not matched. For survival analyses, the Kaplan–Meier method was used to estimate time-to-event probabilities. For all outcomes, hazard ratios (HRs) with corresponding 95% confidence intervals (CIs) and *p*-values and log-rank tests were calculated using Cox proportional hazards model [19]. The proportional hazards assumption was assessed using the generalized Schoenfeld residuals method. More details are provided on the official TriNetX Website. Graphs were created using R (R version 4.5.1, R Foundation for Statistical Computing, Vienna, Austria) and forestplot and ggplot2 packages. The codes for covariates are provided in Table S3.

### 2.5. Use of Artificial Intelligence

ChatGPT-5.1-Modell (OpenAI, San Francisco, CA, USA) was used to assist in formatting data into tables and for language refinement of the manuscript. All data analyses, interpretations, and the final manuscript content were reviewed and validated by the authors. The authors take full responsibility for the integrity and accuracy of the content presented in this publication.

## 3. Results

### 3.1. Identification of PD Patients Treated with SGLT2is

The study identified a total of 19,871 adult PD patients, of which 2.1% (n = 412) initiated SGLT2i therapy within three months after starting PD. The remaining 19,459 patients served as the non-users control group (Figure 1). The median follow-up time (±IQR) was 1.84 (±1.9) years for SGLT2i users and 2.65 (±3.10) years for non-users, respectively. Before PSM, users of SGLT2is were older, compared to non-users (58.7 ± 12.7 years vs. 46.7 ± 19.3 years) and more often male. Furthermore, SGLT2i users exhibited a higher burden of cardiovascular and metabolic risk factors. In parallel to the comorbidities, the medication differed between the groups with higher use of insulin, beta blockers, diuretics and lipid-lowering drugs in the SGLT2i user group (Table 1). Peritonitis rate for users was 2.43% and 1.64% for non-users. We analyzed cohorts before PSM, observing survival rates of 46.39% and 75.15% and MACE rates of 71.5% and 23.6% for SGLT2i users and SGLT2i non-users, respectively. For main analysis, PSM was performed and led to a cohort of 367 patients in each study arm (Table 1, Figure S1). Patients with events prior to the follow-up initiation time were excluded from outcome analyses; therefore, the number of patients for analysis varied between outcomes.

### 3.2. SGLT2is Showed No Significant Association with Mortality and Cardiovascular Events

**Figure 2:**
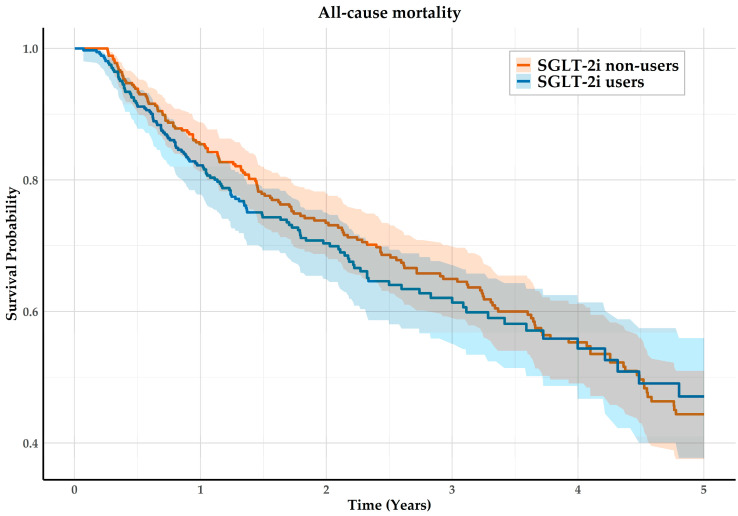
Kaplan–Meier curves for all-cause mortality of SGLT2i users and non-users.

**Figure 3:**
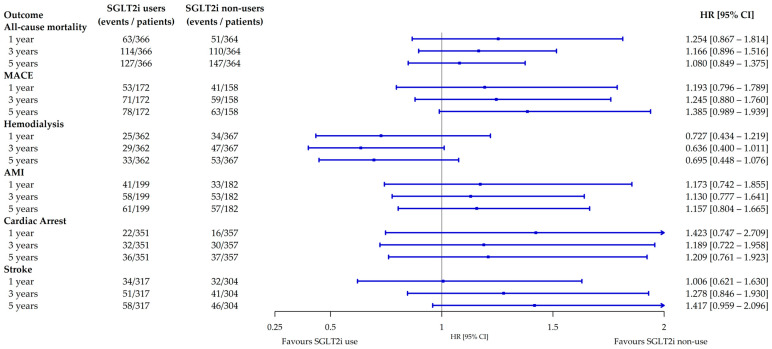
Forest plot of primary and secondary outcomes. 95% CIs represented as error bars. HR hazard ratio; AMI acute myocardial infarction; CI confidence interval; MACE major adverse cardiovascular event; SGLT2i sodium-glucose cotransporter 2 inhibitor.

A total of 274 all-cause mortality events occurred in the matched cohorts, corresponding to a confidence interval width of 0.53. Based on the Schoenfeld approximation, this number of events provides approximately 80% power to detect hazard ratios ≤ 0.71 or ≥1.40. During the follow up, 127 of 366 users (34.7%) and 147 of 364 non-users (40.4%) reached the primary outcome all-cause mortality. In Kaplan–Meier analyses, the estimated 5-year survival probability was 47.1% for SGLT2i users and 44.4% for non-users. SGLT2i use had no significant effect on all-cause mortality (HR 1.08, 95% CI 0.85–1.38; *p* = 0.53; Figure 2 and Figure 3). Similarly, 5-year MACE did not differ significantly (HR 1.39, 95% CI 0.99–1.94; *p* = 0.06). A total of 45.3% for SGLT2i users and 39.9% for non-users reached the secondary outcome. There was no significant effect for individual MACE components (Figure 3). Furthermore, subgroup analyses for 3- and 5-year follow-up showed no significant survival or cardiovascular differences for subgroups with comorbid HF, CKD, or T2DM (Tables S4–S6, Figures S4–S6).

### 3.3. SGLT2is Showed No Increase in Complications in PD Patients

We further assessed renal, metabolic or infectious complications of SGLT2is. Dehydration, metabolic disturbances like ketoacidosis or hypoglycemia and thrombosis showed no significant difference between cohorts. Rates of urinary tract infections were significantly less frequent in the SGLT2i group (Figure S2). Specific PD complications such as peritonitis or catheter problems were not increased with <10 events per group within the follow-up period (exact number censored due to low outcome number).

For transition to HD, we analyzed first instances of HD with 33 of 362 (9.1%) SGLT2i users compared with 52 of 367 (14.4%) patients in the non-user group (Figure S3). This difference did not reach statistical significance (HR 0.70, 95% CI 0.45–1.08; *p* = 0.10).

### 3.4. Sensitivity Analysis Confirmed the Robustness of Findings

To reevaluate our findings, we performed analyses with different follow-up durations of 1, 3 and 5 years after the index event. All-cause mortality and MACE remained consistent across cohorts (Figure 3). To address potential immortal time bias, landmark analyses were conducted and showed consistent results for 5-year all-cause mortality and MACE (Table S7). Additionally, we recalculated our analysis for long-term SGLT2i users: The HRs were 0.814 (95% CI: 0.594–1.115, *p* = 0.1983) for 5-year all-cause mortality and 1.184 (95% CI: 0.778–1.802, *p* = 0.4289) for MACE. For transition to HD, SGLT2i use showed significant reduction with an HR of 0.328 (95% CI: 0.161–0.668, *p* = 0.0013). Exact event numbers for the transition to HD analyses were not publicly available due to censoring (n ≤ 10) (Table S8). When initiating follow-up after 30 days of the index event, we no longer observed significant association with an HR of 0.629 (95% CI: 0.288–1.375, *p* = 0.2413). Regarding continuation of SGLT2is during PD initiation, we did not observe significant association for 5-year mortality, MACE or HD (Table S9).

## 4. Discussion

SGLT2is have shown a broad range of beneficial effects including a reduction in all-cause mortality and MACE in vulnerable patient cohorts including T2DM, CKD and HF patients [1]. Our study provides evidence that these effects are not conserved in PD patients.

### 4.1. No Difference in All-Cause Mortality and MACE

Our analysis did not reveal a significant association of SGLT2i use with all-cause mortality or MACE for patients undergoing PD. This finding contrasts the cardioprotective and survival advantages demonstrated in large randomized controlled trials among patients with earlier stages of CKD and retrospective analyses—similarly to ours—demonstrating benefits in mortality and MACE reduction in diabetic patients with KF initiating dialysis [2,4,20]. The latter, however, included very few PD patients. Regarding continuation of SGLT2i therapy at the initiation of PD, no significant benefit was observed. However, interpretation is limited by small sample size.

It is plausible that the mechanisms mediating these cardiovascular benefits—such as osmotic diuresis, blood pressure reduction, improved ventricular loading conditions, and favorable metabolic effects—are attenuated in PD patients due to severely reduced glomerular filtration and altered volume homeostasis [5,6]. In contrast, a growing body of evidence suggests that SGLT2is benefits are mediated through pleiotropic events. Recent data from a meta-analysis show a reduced relative risk for sudden cardiac death. In addition, direct SGLT2is have positive cellular effects in endothelial cells, cardiomyocytes, and immune cells, although these mechanisms alone may not be sufficient to translate into reductions in mortality or MACE in this high-risk population [21,22].

Notably, our PD cohort was heterogeneous, with SGLT2i users being older and more comorbid. Analysis before PSM showed relevant differences in survival and MACE rate. Similar selection patterns have been reported in real-world data, where SGLT2is are often initiated in patients with greater cardiovascular and metabolic burden despite older age occasionally limiting their use [23]. In summary, our results suggest that once patients progress to KF and receive PD, SGLT2i-mediated cardioprotection may no longer exert clinically relevant effects.

### 4.2. SGLT2is Are Safe in Peritoneal Dialysis Patients

In our cohort, SGLT2i therapy appeared to be safe among patients treated with PD. We observed no increased incidence of adverse events such as urinary tract infections, hypovolemia, or metabolic complications that have typically been associated with this drug class. This is in line with more recent evidence suggesting that infections primarily involve the external genital tract rather than the urinary tract itself [24,25]. With respect to ketoacidosis, the main concern relates to euglycemic ketoacidosis, which differs from classical diabetic ketoacidosis, but is less relevant to the population studied here [26,27].

These results are clinically important because safety concerns have historically limited the use of SGLT2is in advanced CKD [28]. Our data add to the growing body of evidence supporting the overall safety of these agents in KF and specifically in PD patients. This finding is particularly relevant given the paucity of real-world data in this population and the ongoing need for prospective confirmation. Several randomized controlled trials are currently investigating SGLT2i use in PD, including their safety, effects on ultrafiltration, and mechanistic pathways, with results still awaited [13]. Nevertheless, our study contributes valuable preliminary evidence suggesting that SGLT2is can be safely prescribed in PD, while emphasizing the need for larger, prospective studies to validate these observations and define their clinical role in this fragile population.

### 4.3. No Evident Impact on Transition to Hemodialysis

Experimental studies have demonstrated that SGLT2is reduce peritoneal glucose absorption, attenuate peritoneal fibrosis, and enhance ultrafiltration capacity [13]. These findings suggest that SGLT2i therapy may preserve peritoneal membrane integrity and, consequently, improve long-term PD technique survival. Based on this rationale, we investigated whether SGLT2i use was associated with a reduced risk of transition to hemodialysis. However, our analysis did not reveal a significant difference in transition rates between patients receiving SGLT2is and those who were not.

The absence of a detectable signal for PD dropout supports the assumption that SGLT2is do not negatively influence peritoneal membrane function, ultrafiltration capacity, or dialysis adequacy [29,30]. These results are reassuring from a clinical standpoint, indicating that SGLT2i therapy can be safely continued in PD patients without compromising treatment continuity. However, our data also do not suggest a measurable benefit in terms of preserving PD modality or delaying the need for HD. Although SGLT2is have been shown to slow the decline of kidney function in earlier CKD stages, this effect may be negligible once patients are established on PD [4].

Taken together, our findings imply that SGLT2i therapy neither harms nor substantially alters the course of PD treatment. As recruiting patients for prospective studies in PD remains challenging due to the limited number of eligible individuals, our findings provide valuable real-world evidence on clinically meaningful outcomes. Future studies should aim to confirm these results in prospective designs focusing on hard endpoints such as technique failure and mortality. Currently, the Renal Lifecycle trial is investigating the effect of the SGLT2i dapagliflozin in severe CKD, HD and PD patients, for a composite endpoint including all-cause mortality. The results will provide randomized controlled evidence for the questions addressed by our study [31].

### 4.4. Limitations

This study has several limitations related to the retrospective, EHR-based nature: a potential limitation is misclassification bias due to identification of patients through diagnostic, medication and procedural codes. To mitigate this, patients were required to have at least two PD records spaced ≥3 months apart, as used in previous retrospective studies to ensure consistent PD treatment [32]. Still, in a global study, variation in practice of coding must be assumed. Due to the observational design, selection bias might therefore limit the generalizability of our findings. A potential limitation of the study is insufficient power. An effect size comparable to that reported in the DAPA-CKD trial (HR = 0.69) would have been detectable in our dataset, whereas smaller effect sizes would likely not have been identified.

Additionally, the relatively low number of patients on SGLT2is (resulting from lack of approval of SGLT2is in KF) in the dataset did not allow differentiation between modalities of PD, e.g., continuous or intermittent PD. This prevented evaluation of potential differences in outcomes by PD modality. Prescription of SGLT2is is based on documented prescription, administration or dispensing; therefore, discontinuation or intermittent use cannot be ruled out. Furthermore, the TriNetX database does not provide granular clinical data, for example, on ultrafiltration parameters or urine volume, which may represent a clinically relevant benefit for PD patients but remained beyond the scope of this analysis. This further accounts for SGLT2i indications as well as comorbidity subtypes, such as different forms of heart failure.

While the observational and retrospective design of this study entails inherent limitations, the data provide meaningful real-world evidence that complements existing trial results and warrants confirmation in prospective settings.

## 5. Conclusions

In summary, our study provides the first data demonstrating that the benefits of SGLT2is regarding all-cause mortality and MACE do not transfer to PD patients, while safety concerns appear unfounded. Further research should aim to define effective and evidence-based cardio-renal protection for this underserved population.

## Figures and Tables

**Table 1:** Baseline characteristics of cohorts before and after propensity score matching.

Characteristic	SGLT2is Users Before PSM (n = 412)	SGLT2is Non-Users Before PSM (n = 19,459)	SMD Before PSM	SGLT2is Users After PSM (n = 367)	SGLT2is Non-Users After PSM (n = 367)	SMD After PSM
Age at Index	58.7 ± 12.7	46.7 ± 19.3	0.728	58.8 ± 13.1	59.4 ± 14.4	0.04
Not Hispanic or Latino	380 (92.23%)	15,893 (81.67%)	0.317	338 (92.10%)	339 (92.37%)	0.01
Male	264 (64.08%)	7604 (39.08%)	0.517	232 (63.22%)	232 (63.22%)	<0.0001
White	208 (50.49%)	8341 (42.86%)	0.153	189 (51.50%)	194 (52.86%)	0.027
Heart failure	305 (74.03%)	3129 (16.08%)	14,327	261 (71.12%)	260 (70.85%)	0.006
Type 2 diabetes mellitus	256 (62.14%)	3743 (19.24%)	0.971	219 (59.67%)	223 (60.76%)	0.022
Dyslipidemia	231 (56.07%)	3833 (19.70%)	0.809	205 (55.86%)	205 (55.86%)	<0.0001
Hypertension	221 (53.64%)	5737 (29.48%)	0.506	199 (54.22%)	191 (52.04%)	0.044
CIHD	230 (55.83%)	3123 (16.05%)	0.911	195 (53.13%)	193 (52.59%)	0.011
Overweight and obesity	181 (43.93%)	3663 (18.82%)	0.562	161 (43.87%)	162 (44.14%)	0.005
Acute myocardial infarction	169 (41.02%)	1271 (6.53%)	0.886	137 (37.33%)	131 (35.70%)	0.034
Hypertensive heart disease	173 (41.99%)	1029 (5.29%)	0.958	136 (37.06%)	146 (39.78%)	0.056
Cardiomyopathy	126 (30.58%)	905 (4.65%)	0.724	102 (27.79%)	109 (29.70%)	0.042
CLRD	88 (21.36%)	2673 (13.74%)	0.201	82 (22.34%)	74 (20.16%)	0.053
Diseases of liver	70 (16.99%)	1482 (7.62%)	0.288	58 (15.80%)	54 (14.71%)	0.03
Neoplasms	55 (13.35%)	2111 (10.85%)	0.077	53 (14.44%)	10 (2.73%)	0.008
Peritonitis	10 (2.43%)	320 (1.64%)	0.055	10 (2.73%)	257 (70.03%)	<0.0001
Beta blocking agents	311 (75.49%)	5391 (27.70%)	10,886	268 (73.03%)	241 (65.67%)	0.066
Diuretics	305 (74.03%)	4451 (22.87%)	11,915	263 (71.66%)	202 (55.04%)	0.13
Statins	266 (64.56%)	3744 (19.24%)	10,341	229 (62.40%)	185 (50.41%)	0.034
Insulins	248 (60.19%)	3324 (17.08%)	0.987	215 (58.58%)	117 (31.88%)	0.072
RASi	232 (56.31%)	3460 (17.78%)	0.87	196 (53.41%)	36 (9.81%)	0.06
Calcium channel blockers	156 (37.86%)	3289 (16.90%)	0.484	137 (37.33%)	14 (3.82%)	0.115
Biguanides	55 (13.35%)	764 (3.93%)	0.34	46 (12.53%)	15 (4.09%)	0.087
Sulfonylureas	22 (5.34%)	463 (2.38%)	0.154	22 (6.00%)	11 (3.00%)	0.101
DPP-4 inhibitors	22 (5.34%)	254 (1.31%)	0.227	20 (5.45%)	132 (35.97%)	0.064
GLP-1 analogues	17 (4.13%)	183 (0.94%)	0.204	15 (4.09%)	75 (20.44%)	0.059
GFR mL/min/1.73 m^2^	65.4 ± 33.5	71.2 ± 52.5	0.133	64.6 ± 33.9	61 ± 36	0.097
GFR 0–15 mL/min/1.73 m^2^	141 (34.22%)	7503 (38.56%)	0.09	138 (37.60%)	142 (38.69%)	0.034
GFR 15–30 mL/min/1.73 m^2^	85 (20.63%)	1461 (7.51%)	0.384	74 (20.16%)	157 (42.78%)	0.007
GFR 30–45 mL/min/1.73 m^2^	161 (39.08%)	1958 (10.06%)	0.716	140 (38.15%)	213 (58.04%)	0.011
GFR 45–60 mL/min/1.73 m^2^	190 (46.12%)	2547 (13.09%)	0.776	160 (43.60%)	157 (42.78%)	0.017
Potassium, mmol/L	4.04 ± 0.5	4.0 ± 0.6	0.072	4.05 ± 0.5	4.04 ± 0.5	0.019
SBP, mmHg	105 ± 25.4	121 ± 22.5	0.653	106 ± 24.9	113.6 ± 28.1	0.258
DBP, mmHg	62 ± 16.1	71 ± 14.1	0.576	63 ± 15.7	65 ± 15.3	0.139
BMI	30.2 ± 7.8	30.7 ± 7.7	0.076	30.5 ± 7.7	31.3 ± 8.1	0.098
Albumin, g/dL	3.4 ± 0.5	3.5 ± 0.6	0.157	3.5 ± 0.5	3.5 ± 0.5	0.151
AST, U/L	34.9 ± 44.3	35.5 ± 20.0	0.004	34.7 ± 4.5	31 ± 36	0.089
ALT, U/L	31.0 ± 3.8	31 ± 15.2	0.009	30.0 ± 35.3	25.6 ± 28.4	0.136
Hemoglobin A1c, %	7.4 ± 2.2	6.7 ± 1.9	0.339	7.4 ± 2.2	7.3 ± 2	0.062
BNP, pg/mL	808 ± 852	584 ± 1274	0.207	766 ± 829	792 ± 915	0.03
TG, mg/dL	143 ± 183	160 ± 246	0.079	147 ± 198	157 ± 162	0.054
HDL, mg/dL	38.3 ± 15.1	43.3 ± 18.1	0.299	38.3 ± 15.3	39.2 ± 15.7	0.056
LDL, mg/dL	81.8 ± 38.1	90.4 ± 41.3	0.217	80.4 ± 36.7	88.4 ± 41.2	0.207

SGLT2i, sodium-glucose cotransporter 2 inhibitor; PSM, propensity score matching; SMD, standardized mean difference; CIHD, chronic ischemic heart disease; CLRD, chronic lower respiratory disease; BMI, body mass index; GFR, glomerular filtration rate; SBP, systolic blood pressure; DBP, diastolic blood pressure; AST, aspartate aminotransferase; ALT, alanine aminotransferase; BNP, B-type natriuretic peptide; TG, triglycerides; HDL, high-density lipoprotein; LDL, low-density lipoprotein; RASi, renin–angiotensin system inhibition; DPP-4, dipeptidyl peptidase-4; GLP-1, glucagon-like peptide-1.

## Data Availability

The original contributions presented in this study are included in the article/Supplementary Material. Further inquiries can be directed to the corresponding author(s).

## Supplementary Materials

The following supporting information can be downloaded at https://www.mdpi.com/article/10.3390/jcm14248815/s1, Table S1. Codes and cohort query definitions. Table S2. Definitions of study outcomes. Table S3. Propensity score matching (PSM) codes. Table S4. Main outcomes in chronic kidney disease (CKD). Table S5. Main outcomes in type 2 diabetes (T2DM). Table S6. Main outcomes in heart failure (HF). Table S7. Landmark analysis. Table S8. Main outcomes in long-term SGLT2is. Table S9. SGLT2is continuation outcome analysis. Figure S1. Balance plot for PSM. Figure S2. Forest plot of safety outcomes. Figure S3 Kaplan–Meier curves for hemodialysis. Figure S4. Forest plot of outcomes for CKD. Figure S5. Forest plot of outcomes for HF. Figure S6. Forest plot of safety type 2 diabetes. STROBE Checklist.

## Abbreviations

The following abbreviations are used in this manuscript:

SGLT2i	Sodium-glucose cotransporter 2 inhibitor
PD	Peritoneal dialysis
HD	Hemodialysis
KF	Kidney failure
KRT	Kidney replacement therapy
HF	Heart failure
CKD	Chronic kidney disease
T2DM	Type 2 diabetes mellitus
EHR	Electronic health records
PSM	Propensity score matching
MACE	Major adverse cardiovascular events
SNOMED	Systematized Nomenclature of Medicine
ICD-10	International Classification of Diseases, 10th Revision
HCO	Health care organization
IQR	Interquartile range
SD	Standard deviation
CI	Confidence interval
HR	Hazard ratio
CIHD	Chronic ischemic heart disease
CLRD	Chronic lower respiratory disease
BMI	Body mass index
GFR	Glomerular filtration rate
SBP	Systolic blood pressure
DBP	Diastolic blood pressure
BNP	B-type natriuretic peptide
TG	Triglycerides
HDL	High-density lipoprotein
LDL	Low-density lipoprotein
AST	Aspartate aminotransferase
ALT	Alanine aminotransferase
RASi	Renin–angiotensin system inhibition
HbA1c	Hemoglobin A1c
STROBE	Strengthening the Reporting of Observational Studies in Epidemiology
