# Supplementary material for: Sodium-Glucose Cotransporter 2 Inhibitor Use in Adults Undergoing Peritoneal Dialysis: A Propensity-Matched Real-World Data Analysis
Source: J Clin Med. 2025 Dec 12;14(24):8815. doi: 10.3390/jcm14248815 (PMC12733463; doi:10.3390/jcm14248815)
Supplement: Supplementary file 1 [file jcm-14-08815-s001.zip › jcm-4029624-supplementary.pdf]

Sodium-glucose cotransporter 2 inhibitor use in adults undergoing peritoneal dialysis: A propensity-matched real-world data analysis

List of Supplementary Files

Supplementary Table S1. Codes and cohort query definitions.

Supplementary Table S2. Definition of study outcomes.

Supplementary Table S3. Propensity score matching codes.

Supplementary Table S4. Main outcomes in chronic kidney disease

Supplementary Table S5. Main outcomes in type 2 diabetes

Supplementary Table S6. Main outcomes in heart failure

Supplementary Table S7. Landmark analysis

Supplementary Table S8. Main outcomes in long-term SGLT2i

Supplementary Table S9. SGLT2i continuation outcome analysis.

Supplementary Figure S1. Balance plot for PSM

Supplementary Figure S2. Forest plot of safety outcomes

Supplementary Figure S3. Kaplan-Meier curves for transition to hemodialysis of SGLT2i users and non-users

Supplementary Figure S4. Forest plot of outcomes for CKD

Supplementary Figure S5. Forest plot of outcomes for HF

Supplementary Figure S6. Forest plot of safety type 2 diabetes

STROBE Checklist

Supplementary Table S1. Demographic, procedural, and medication codes used in the definition of the cohorts.

**Category**	**Code**	**Description**
Time Limitation: 01.01.2015 - 01.01.2025		
Demographics	Age	Age (≥ 18 years)
#**1**: Peritoneal Dialysis (first instance)		
Procedure	SNOMED:1172536000	Peritoneal Dialysis
Procedure	SNOMED: 71192002	Peritoneal Dialysis
Medication	VA: IR200	Peritoneal Dialysis Solution
#**2:** SGLT2 inhibitor (within 1 year after the first instance of #1)		
Medication	NLM: ATC: A10BK	Sodium-glucose co-transporter 2 (SGLT2) inhibitors
#**3**: Peritoneal Dialysis (second instance; at least 3 months after the first instance of PD)		
Procedure	SNOMED:1172536000	Peritoneal Dialysis
Procedure	SNOMED: 71192002	Peritoneal Dialysis
Medication	VA: IR200	Peritoneal Dialysis Solution
#**4**: Exclusion of Hemodialysis		
Procedure	SNOMED:302497006	Hemodialysis
Procedure	CPT: 90937	Hemodialysis procedure requiring repeated evaluation(s) with or without substantial revision of dialysis prescription
Medication	CPT: 90935	Hemodialysis procedure with single evaluation by a physician or other qualified health care professional

Procedure	CPT:1012752	Hemodialysis Procedures
#5: Codes for subgroups		
Diagnoses	ICD-10-CM: N18	Chronic Kidney Disease
Diagnoses	ICD-10-CM: I50	Heart failure
Diagnoses	ICD-10-CM: E11	Type 2 diabetes mellitus

SNOMED, Systematized Nomenclature of Medicine; VA IR200, Veterans Affairs (Pharmacy Code) for Peritoneal Dialysis Solution; NLM, National Library of Medicine; ATC, Anatomical Therapeutic Chemical Classification System; CPT, Current Procedural Terminology; ICD-10-CM, International Classification of Diseases, 10th Revision, Clinical Modification; PD, Peritoneal Dialysis; SGLT2, Sodium-Glucose Co-Transporter 2;

Supplementary Table S2. Definition of study outcomes.

Outcome	Category	Code	Description
**Mortality**	Diagnosis	ICD-10-CM: R99	Ill-defined and unknown cause of mortality
	Demographics	Deceased	Deceased
**MACE**	Diagnosis	ICD-10-CM: I21	Acute myocardial infarction
	Diagnosis	ICD-10-CM: I63	Cerebral infarction
	Diagnosis	ICD-10-CM: I61	Nontraumatic intracerebral hemorrhage
	Diagnosis	ICD-10-CM: I46	Cardiac arrest
**Hemodialysis**	CPT	90937	Hemodialysis procedure requiring repeated evaluation(s)
	CPT	1812752	Hemodialysis Procedures
	CPT	90935	Hemodialysis procedure with single evaluation
	SNOMED	302497006	Hemodialysis
**Ketoacidosis**	Diagnosis	ICD-10-CM: E11.1	Type 2 diabetes mellitus with ketoacidosis
	Diagnosis	ICD-10-CM: E13.1	Other specified diabetes mellitus with ketoacidosis
	Diagnosis	ICD-10-CM: E10.1	Type 1 diabetes mellitus with ketoacidosis
**Cardiac arrest**	Diagnosis	ICD-10-CM: I46	Cardiac arrest
**Stroke**	Diagnosis	ICD-10-CM: I61	Nontraumatic intracerebral hemorrhage
	Diagnosis	ICD-10-CM: I63	Cerebral infarction
**Acute myocardial infarction (AMI)**	Diagnosis	ICD-10-CM: I21	Acute myocardial infarction
**Genitourinary Infection (UTI)**	Diagnosis	ICD-10-CM: N39.0	Urinary tract infection, site not specified
	Diagnosis	ICD-10-CM: N30	Cystitis
	Diagnosis	ICD-10-CM: N10	Acute pyelonephritis
	Diagnosis	ICD-10-CM: B37.3	Candidiasis of vulva and vagina
	Diagnosis	ICD-10-CM: B37.4	Candidiasis of other urogenital sites
**Hypoglycemia**	Diagnosis	ICD-10-CM: E16.2	Hypoglycemia, unspecified
	Lab Value	TNX: 9825	Glucose (≤ 70 mg/dL)
	Lab Value	LOINC: 2345-7	Glucose in Serum or Plasma (≤ 70 mg/dL)

**Peritonitis**	Diagnosis	ICD-10-CM: K65	Peritonitis
**PD Catheter issues**	Diagnosis	ICD-10-CM: T85.691A	Mechanical complication of dialysis catheter
	Diagnosis	ICD-10-CM: T85.71XA	Infection due to peritoneal dialysis catheter
**Osteoporotic fracture**	Diagnosis	ICD-10-CM: M80	Osteporotic fracture
**Thrombosis**	Diagnosis	ICD-10-CM: I82	Arterial embolism and thrombosis
	Diagnosis	ICD-10-CM: I26	Pulmonary embolism
	Diagnosis	ICD-10-CM: I74	Other venous embolism and thrombosis
**Below-knee amputation**	Diagnosis	ICD-10-CM: Z89.41	Acquired absence of great toe
	Diagnosis	ICD-10-CM: Z89.42	Acquired absence of other toe(s)
	Diagnosis	ICD-10-CM: Z89.43	Acquired absence of foot
	Diagnosis	ICD-10-CM: Z89.5	Acquired absence of leg below knee
**Dehydration**	Diagnosis	ICD-10-CM: E86.0	Dehydration
**Hypervolemia**	Diagnosis	ICD-10-CM: E86.0	Fluid overload

ICD-10-CM, International Classification of Diseases, 10th Revision Clinical Modification; CPT, Current Procedural Terminology; SNOMED Systematized Nomenclature of Medicine; LOINC, Logical Observation Identifiers Names and Codes; TNX, TriNetX curated

Supplementary Table S3. Propensity score matching codes.

Source	Code	Description
AI	Age at Index	Age
M	Male	White
LOINC	White	Race
LOINC	Not Hispanic or Latino	Ethnicity
ATC	C07	Beta blocking agents
ATC	C03	Diuretics
ATC	C10AA	HMG CoA reductase inhibitors
ATC	A10A	Insulins and analogues
ATC	C09	Agents acting on the renin-angiotensin system
ATC	C08	Calcium channel blockers
ATC	A10BA	Biguanides
ATC	A10BB	Sulfonylureas
ATC	A10BH	Dipeptidyl peptidase 4 (DPP-4) inhibitors
ATC	A10BJ	Glucagon-like peptide-1 (GLP-1) analogues
TNX Curated	9028 Potassium [Moles/volume] in Serum	Plasma or Blood Potassium
TNX Curated	9085 Blood Pressure	Systolic Blood Pressure
TNX Curated	9086 Blood Pressure	Diastolic Blood Pressure
TNX Curated	8001 Glomerular filtration rate/1.73 sq M.predictedin Serum	Plasma or Blood by Creatinine-based formula (MDRD)
TNX Curated	9083 BMI	
TNX Curated	9045 Albumin [Mass/volume] in Serum	Plasma or Blood
TNX Curated	9047 Aspartate aminotransferase [Enzymatic activity/volume]	
TNX Curated	9044 Alanine aminotransferase [Enzymatic activity/volume]	Plasma or Blood
TNX Curated	9037 Hemoglobin A1c/Hemoglobin.total in Blood	
TNX Curated	9003 Natriuretic peptide B [Mass/volume] in Serum	Plasma or Blood
TNX Curated	9004 Triglyceride [Mass/volume] in Serum	Plasma or Blood
TNX Curated	9001 Cholesterol in HDL [Mass/volume] in Serum or Plasma	

TNX Curated	9002 Cholesterol in LDL [Mass/volume] in Serum or Plasma	
ICD-10-CM	I50	Heart failure
ICD-10-CM	E11	Type 2 diabetes mellitus
ICD-10-CM	E78	Dyslipidemia
ICD-10-CM	I10	Essential (primary) hypertension
ICD-10-CM	I25	Chronic ischemic heart disease
ICD-10-CM	E66	Overweight and obesity
ICD-10-CM	I21	Acute myocardial infarction
ICD-10-CM	I11	Hypertensive heart disease
ICD-10-CM	I42	Cardiomyopathy
ICD-10-CM	J40-J4A	Chronic lower respiratory diseases
ICD-10-CM	K70-K77	Diseases of liver
ICD-10-CM	C00-D49	Neoplasms
ICD-10-CM	K65	Peritonitis

ICD-10-CM, International Classification of Diseases, 10th Revision Clinical Modification; ATC, Anatomical Therapeutic Chemical Classification System; SNOMED Systematized Nomenclature of Medicine; LOINC, Logical Observation Identifiers Names and Codes; TNX, TriNetX curated

Supplementary Table S4. Main outcomes in chronic kidney disease

	SGLT-2i users	SGLT2i non-users	HR	p-value (Log-Rank-Test)
	137	136		
**All-cause mortality**				
3 years	51/137	39/136	1.482 (0.974, 2.255)	0.0641
5 years	51/137	51/136	1.275 (0.858, 1.894)	0.2284
**MACE**				
3 years	25/63	14/52	1.883 (0.973, 3.645)	0.0562
5 years	25/63	15/52	1.794 (0.939,3.428)	0.0727
**Hemodialysis**				
3 years	18/133	19/135	0.982 (0.515, 1.872)	0.9553
5 years	19/133	21/135	1.012 (0.54, 1.895)	0.9705
**AMI**				
3 years	17/73	12/61	1.408 (0.669, 2.962)	0.3648
5 years	17/73	14/61	1.298 (0.632, 2.665)	0.4760
**Cardiac Arrest**				
3 years	/	/	/	/
5 years	/	/	/	/
**Stroke**				
3 years	19/116	18/117	1.215 (0.635, 2.325)	0.5561
5 years	19/116	19/117	1.166 (0.614, 2.213)	0.6383

Abbreviation: HR, hazard ratio; CI, confidence interval; MACE, major adverse cardiovascular events; SGLT-2i, sodium–glucose cotransporter 2 inhibitors; AMI, acute myocardial infarction

Events for cardiac arrest were censored due to low outcome numbers (<10)

Supplementary Table S5. Main outcomes in type 2 diabetes

	SGLT-2i users	SGLT2i non-users	HR	p-value (Log-Rank-Test)
	154	154		
**All-cause mortality**				
3 years	40/154	53/154	0.798 (0.529, 1.203)	0.2803
5 years	45/154	64/154	0.791 (0.539, 1.159)	0.2279
**MACE**				
3 years	28/80	23/60	0.938 (0.53, 1.629	0.8197
5 years	32/80	26/60	1.07 (0.632, 1.811)	0.8019
**Hemodialysis**				
3 years	/	/	/	/
5 years	11/154	22/154	0.542 (0.262, 1.121)	0.0936
**AMI**				
3 years	22/82	18/71	1.13 (0.605, 2.109)	0.7012
5 years	25/85	20/71	1.271 (0.701, 2.303)	0.4287
**Cardiac Arrest**				
3 years	/	/	/	/
5 years	11/151	12/148	1.106 (0.486, 2.518)	0.8105
**Stroke**				
3 years	18/138	23/127	0.71 (0.383, 1.316)	0.2749
5 years	21/138	26/127	0.759 (0.426, 1.35)	0.3459

Abbreviation: HR, hazard ratio; CI, confidence interval; MACE, major adverse cardiovascular events; SGLT-2i, sodium–glucose cotransporter 2 inhibitors; AMI, acute myocardial infarction

Events for cardiac arrest and hemodialysis were censored due to low outcome numbers (<10)

Supplementary Table S6. Main outcomes in heart failure

	SGLT-2i users	SGLT2i non-users	HR	p-value (Log-Rank-Test)
	218	217		
**All-cause mortality**				
3 years	60/218	78/217	0.882 (0.629, 1.237)	0.4657
5 years	64/218	95/217	0.874 (0.634, 1.205)	0.4105
**MACE**				
3 years	46/104	33/64	0.864 (0.551, 1.354)	0.5235
5 years	48/104	39/72	0.882 (0.576, 1.352)	0.5662
**Hemodialysis**				
3 years	21/215	29/218	0.746 (0.426, 1.309)	0.3058
5 years	25/215	35/218	0.809 (0.482, 1.359)	0.4224
**AMI**				
3 years	34/111	24/73	1.023 (0.604, 1.73)	0.933
5 years	35/111	30/73	0.972 (0.594, 1.592)	0.9110
**Cardiac Arrest**				
3 years	21/212	25/205	0.903 (0.505, 1.615)	0.7310
5 years	24/212	29/205	0.972 (0.563, 1.678)	0.9198
**Stroke**				
3 years	26/189	38/185	0.707 (0.429, 1.166)	0.1725
5 years	26/189	40/185	0.742 (0.451, 1.221)	0.2389

Abbreviation: HR, adjusted hazard ratio; CI, confidence interval; MACE, major adverse cardiovascular events; SGLT-2i, sodium–glucose cotransporter 2 inhibitors; AMI, acute myocardial infarction

Supplementary Table S7. Landmark analysis

	SGLT2i users	SGLT2i non-users	HR	p-value (Log-Rank-Test)
	367	367		
**Follow-up initiation day 14**				
all-cause mortality	109/365	101/165	1.247 (0.95, 1.636)	0.1107
MACE	65/165	54/156	1.412 (0.982, 2.031)	0.0617
**Follow-up initiation day 30**				
all-cause mortality	108/364	101/365	1.236 (0.942, 1.623)	0.1262
MACE	60/160	51/153	1.401 (0.962, 2.04)	0.0776
**Follow-up initiation day 60**				
all-cause mortality	108/364	101/365	1.236 (0.942,1.623)	0.1262
MACE	53/153	46/148	1.408 (0.946,2.096)	0.0902
**Follow-up initiation day 90**				
all-cause mortality	102/358	107/366	1.075 (0.82, 1.411)	0.6004
MACE	52/152	45/152	1.444 (0.966, 2.158)	0.0716
**Follow-up initiation day 180**				
all-cause mortality	76/332	85/349	1.07 (0.785, 1.46)	0.6687
MACE	35/135	33/135	1.426 (0.883, 2.302)	0.1443

Abbreviation: HR, hazard ratio; CI, confidence interval; MACE, major adverse cardiovascular events; SGLT-2i, sodium–glucose cotransporter 2 inhibitors

Supplementary Table S8. Main outcomes in long-term SGLT2i

	SGLT2i users	SGLT2i non-users	HR	p-value (Log-Rank-Test)
	235	235		
**All-cause mortality**				
1 year	28/234	28/232	0.981 (0.581, 1.656)	0.9415
3 years	58/234	67/232	0.959 (0.674, 1.365)	0.8171
5 years	67/234	96/232	0.814 (0.594, 1.115)	0.1983
**MACE**				
1 years	27/104	20/92	1.273 (0.714, 2.269)	0.4124
3 years	46/108	35/100	1.368 (0.879, 2.129)	0.1634
5 years	48/108	41/100	1.184 (0.778, 1.802)	0.4289
**Hemodialysis**				
1 years	/	/	/	/
3 years	/	/	/	/
5 years	<10/235	33/235	0.328 (0.161,0.668)	0.0013

Abbreviation: HR, hazard ratio; CI, confidence interval; MACE, major adverse cardiovascular events; SGLT2i, sodium–glucose cotransporter 2 inhibitors

Events for hemodialysis were censored due to low outcome numbers (<10)

Supplementary Table S9. SGLT2i continuation outcome analysis.

	**SGLT2i users**	**SGLT2i non-users**	**HR**	**p-value (Log-Rank-Test)**
	89	89		
All-cause mortality	35/89	29/89	1.402 (0.854, 2.302)	0.1794
MACE	24/47	23/47	1.205 (0.678,2.14)	0.5239
Hemodialysis	/	/	/	/

Abbreviation: HR, hazard ratio; CI, confidence interval; MACE, major adverse cardiovascular events; SGLT-2i, sodium–glucose cotransporter 2 inhibitors

Events for hemodialysis were censored due to low outcome numbers (<10)

Supplementary Figure S1. Forest plot of safety outcomes

Outcome	SGLT2i users (events / patients)	SGLT2i non-users (events / patients)	HR [95% CI]
**Ketoacidosis**			
3 years	12/347	13/357	1.110 [0.505 – 2.441]
5 years	14/347	16/357	1.220 [0.588 – 2.535]
**Thrombosis**			
1 year	48/296	49/293	0.964 [0.647 – 1.435]
3 years	74/296	70/293	1.139 [0.821 – 1.582]
5 years	82/296	79/293	1.250 [0.912 – 1.711]
**Below-knee amputation**			
3 years	11/348	15/345	0.803 [0.368 – 1.752]
5 years	11/348	20/345	0.697 [0.330 – 1.473]
**Urinary tract infection (UTI)**			
1 year	39/299	45/262	0.739 [0.481 – 1.135]
3 years	55/299	75/262	0.676 [0.477 – 0.958]
5 years	59/299	87/262	0.678 [0.486 – 0.946]
**Dehydration**			
1 year	27/322	34/305	0.749 [0.452 – 1.241]
3 years	41/322	56/305	0.768 [0.513 – 1.151]
5 years	45/322	69/305	0.771 [0.527 – 1.129]
**Hypoglycemia**			
1 year	82/258	72/229	1.028 [0.749 – 1.411]
3 years	112/258	92/229	1.205 [0.914 – 1.590]
5 years	120/258	106/229	1.253 [0.960 – 1.636]

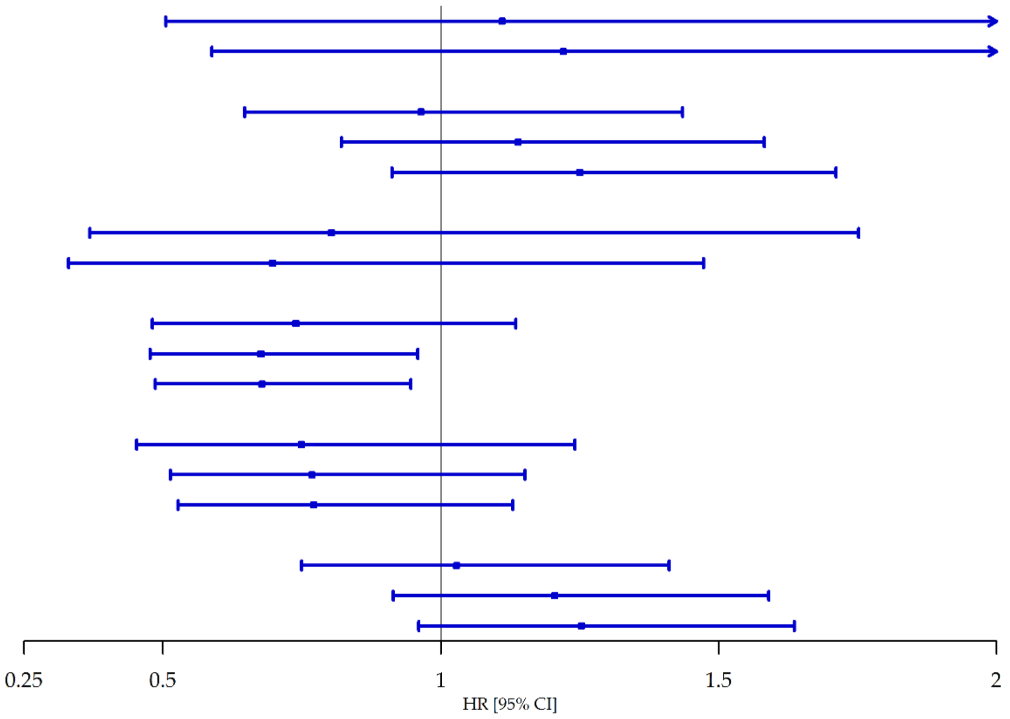

Favours SGLT2i use

Favours SGLT2i non-use

CIs represented as error bars. HR hazard ratio; CI confidence interval; MACE major adverse cardiovascular event; SGLT2i sodium-glucose cotransporter 2 inhibitor; the 1 year outcomes for ketoacidosis were censored due to low outcome numbers (<10)

Supplementary Figure S2. Kaplan-Meier curves for transition to hemodialysis of SGLT2i users and non-users

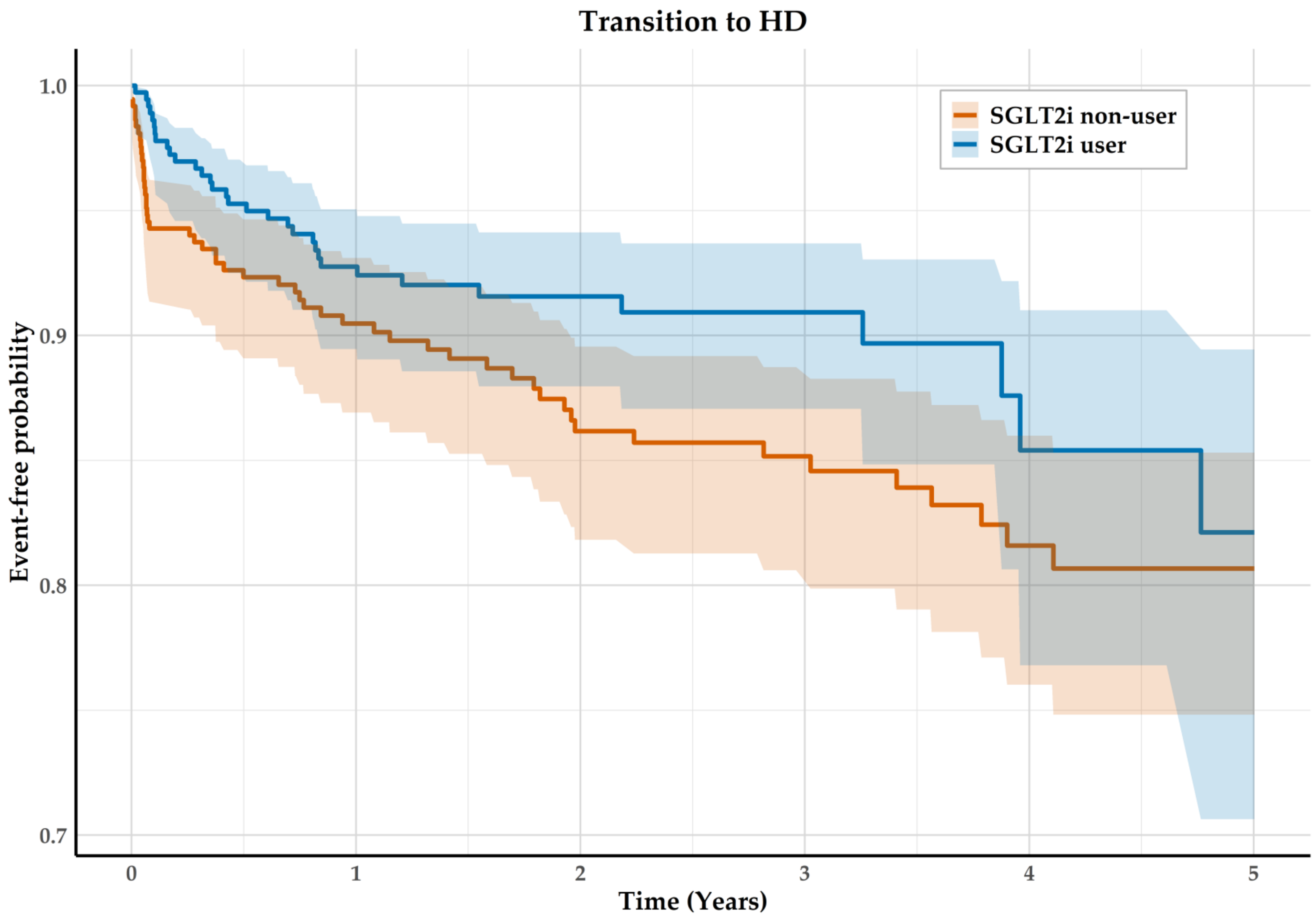

Supplementary Figure S3. Balance plot for PSM

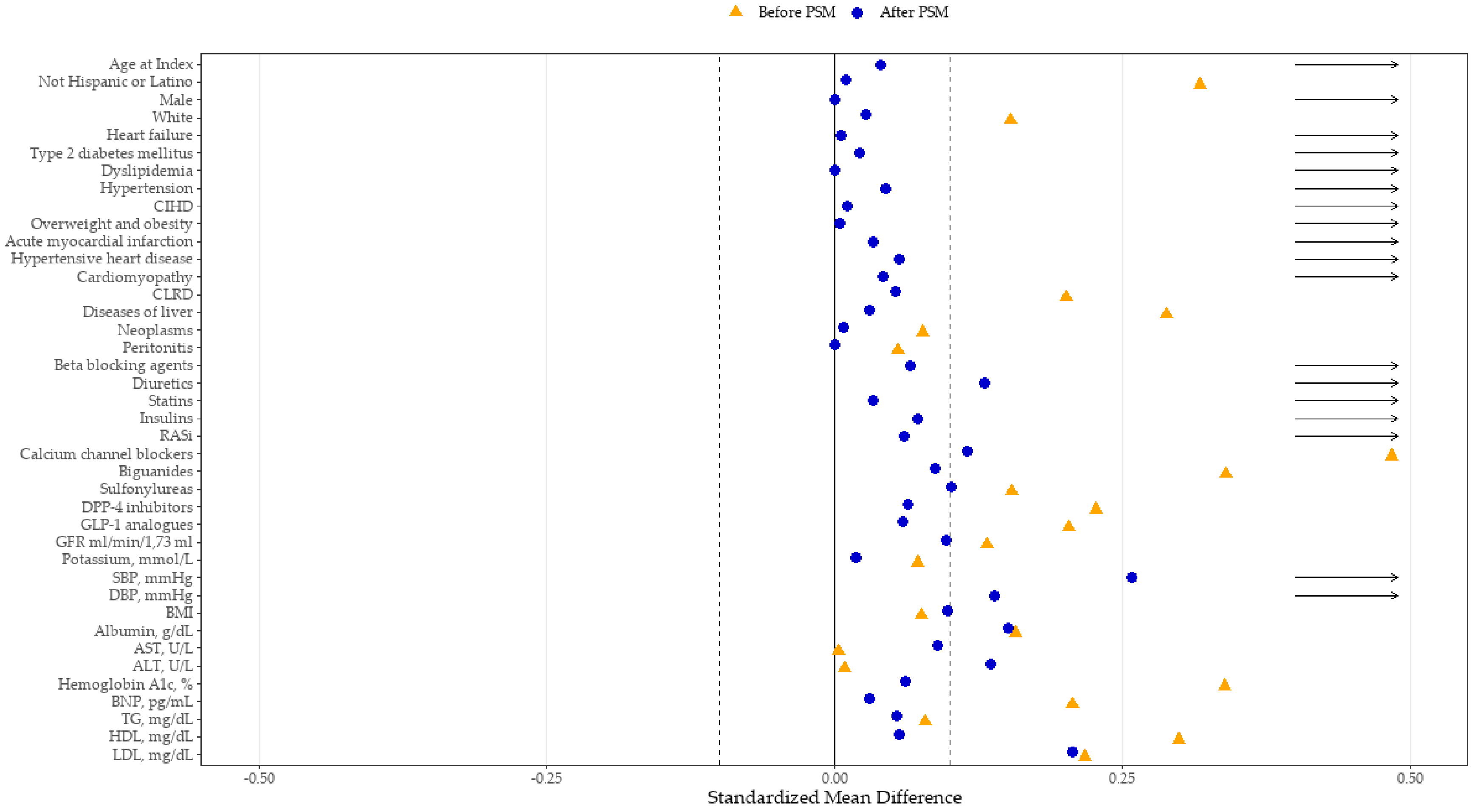

PSM, propensity score matching; CIHD, chronic ischemic heart disease; CLRD, chronic lower respiratory disease; BMI, body mass index; GFR, glomerular filtration rate; SBP, systolic blood pressure; DBP, diastolic blood pressure; AST, aspartate aminotransferase; ALT, alanine aminotransferase; BNP, B-type natriuretic peptide; TG, triglycerides; HDL, high-density lipoprotein; LDL, low-density lipoprotein; RASi, renin–angiotensin system inhibition; DPP-4, dipeptidyl peptidase-4; GLP-1, glucagon-like peptide-1.

Supplementary Figure S4. Forest plot of outcomes for CKD

Outcome	SGLT2i users (events / patients)	SGLT2i non-users (events / patients)	HR [95% CI]
**All-cause mortality**			
3 years	51/137	39/136	1.482 [0.974 – 2.255]
5 years	51/137	51/136	1.275 [0.858 – 1.894]
**MACE**			
3 years	25/63	14/52	1.883 [0.973 – 3.645]
5 years	25/63	15/52	1.794 [0.939 – 3.428]
**Hemodialysis**			
3 years	18/133	19/135	0.982 [0.515 – 1.872]
5 years	19/133	21/135	1.012 [0.540 – 1.895]
**AMI**			
3 years	17/73	12/61	1.408 [0.669 – 2.962]
5 years	17/73	14/61	1.298 [0.632 – 2.665]
**Stroke**			
3 years	19/116	18/117	1.215 [0.635 – 2.325]
5 years	19/116	19/117	1.166 [0.614 – 2.213]

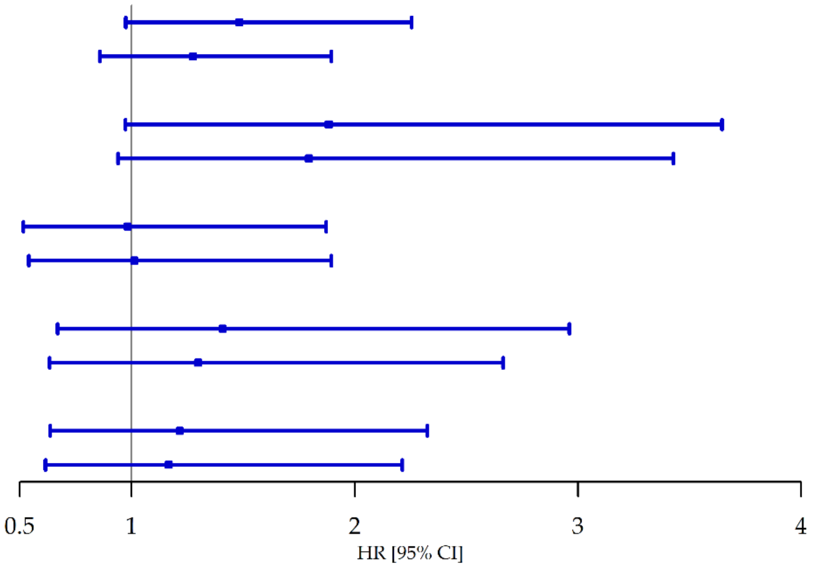

CIs represented as error bars. HR hazard ratio; CI confidence interval; MACE major adverse cardiovascular event; AMI acute myocardial infarction; SGLT2i sodium-glucose cotransporter 2 inhibitor

Supplementary Figure S5. Forest plot of outcomes for HF

Outcome	SGLT2i users (events / patients)	SGLT2i non-users (events / patients)	HR [95% CI]
**All-cause mortality**			
3 years	60/218	78/217	0.882 [0.629 – 1.237]
5 years	64/218	95/217	0.874 [0.634 – 1.205]
**MACE**			
3 years	46/104	33/64	0.864 [0.551 – 1.354]
5 years	48/104	39/72	0.882 [0.576 – 1.352]
**Hemodialysis**			
3 years	21/215	29/218	0.746 [0.426 – 1.309]
5 years	25/215	35/218	0.809 [0.482 – 1.359]
**AMI**			
3 years	34/111	24/73	1.023 [0.604 – 1.730]
5 years	35/111	30/73	0.972 [0.594 – 1.592]
**Cardiac Arrest**			
3 years	21/212	25/205	0.903 [0.505 – 1.615]
5 years	24/212	29/205	0.972 [0.563 – 1.678]
**Stroke**			
3 years	26/189	38/185	0.707 [0.429 – 1.166]
5 years	26/189	40/185	0.742 [0.451 – 1.221]

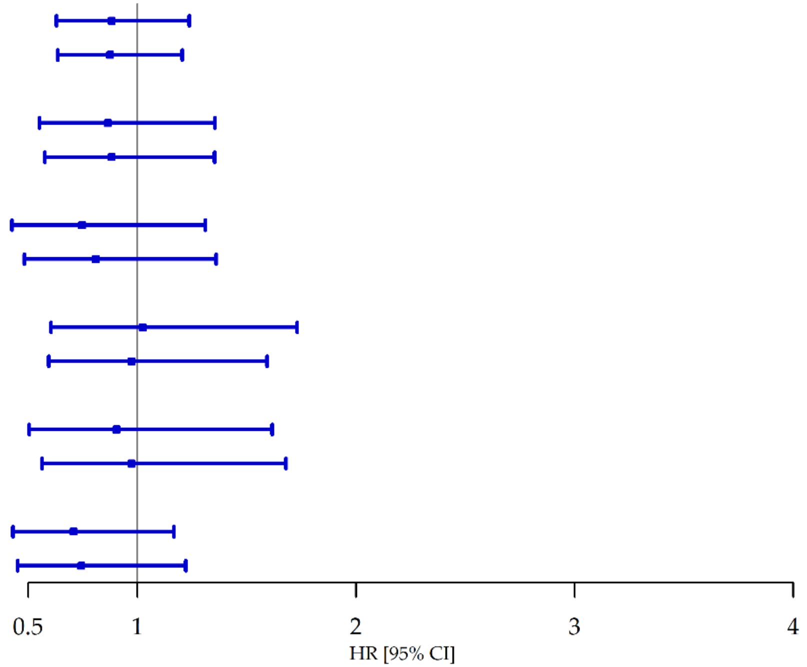

CIs represented as error bars. HR hazard ratio; CI confidence interval; MACE major adverse cardiovascular event; AMI acute myocardial infarction; SGLT2i sodium-glucose cotransporter 2 inhibitor

Supplementary Figure S6. Forest plot of safety type 2 diabetes

Outcome	SGLT2i users (events / patients)	SGLT2i non-users (events / patients)	HR [95% CI]
**All-cause mortality**			
3 years	40/154	53/154	0.798 [0.529 – 1.203]
5 years	45/154	64/154	0.791 [0.539 – 1.159]
**MACE**			
3 years	28/80	23/60	0.938 [0.530 – 1.629]
5 years	32/80	26/60	1.070 [0.632 – 1.811]
**Hemodialysis**			
3 years	/	/	
5 years	11/154	22/154	0.542 [0.262 – 1.121]
**AMI**			
3 years	22/82	18/71	1.130 [0.605 – 2.109]
5 years	25/85	20/71	1.271 [0.701 – 2.303]
**Cardiac Arrest**			
3 years	/	/	
5 years	11/151	12/148	1.106 [0.486 – 2.518]
**Stroke**			
3 years	18/138	23/127	0.710 [0.383 – 1.316]
5 years	21/138	26/127	0.759 [0.426 – 1.350]

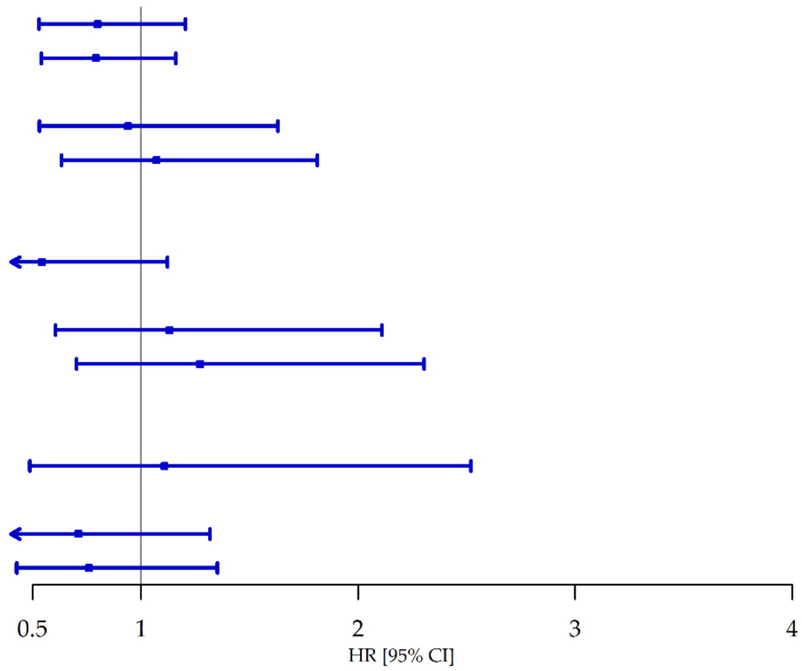

CIs represented as error bars. HR hazard ratio; CI confidence interval; MACE major adverse cardiovascular event; AMI acute myocardial infarction; SGLT2i sodium-glucose cotransporter 2 inhibitor

HR [95% CI]

STROBE Statement—Checklist of items that should be included in reports of ***cohort studies***

	Item No	Recommendation	Page
**Title and abstract**	1	(*a*) Indicate the study's design with a commonly used term in the title or the abstract	1
		(*b*) Provide in the abstract an informative and balanced summary of what was done and what was found	1
**Introduction**			
Background/rationale	2	Explain the scientific background and rationale for the investigation being reported	2
Objectives	3	State specific objectives, including any prespecified hypotheses	2
**Methods**			
Study design	4	Present key elements of study design early in the paper	2/3
Setting	5	Describe the setting, locations, and relevant dates, including periods of recruitment, exposure, follow-up, and data collection	2
Participants	6	(*a*) Give the eligibility criteria, and the sources and methods of selection of participants. Describe methods of follow-up	2/3
		(*b*) For matched studies, give matching criteria and number of exposed and unexposed	3
Variables	7	Clearly define all outcomes, exposures, predictors, potential confounders, and effect modifiers. Give diagnostic criteria, if applicable	3

Data sources/ measurement	8*	For each variable of interest, give sources of data and details of methods of assessment (measurement). Describe comparability of assessment methods if there is more than one group	*3*
Bias	9	Describe any efforts to address potential sources of bias	3
Study size	10	Explain how the study size was arrived at	4
Quantitative variables	11	Explain how quantitative variables were handled in the analyses. If applicable, describe which groupings were chosen and why	3
Statistical methods	12	(*a*) Describe all statistical methods, including those used to control for confounding	3
		(*b*) Describe any methods used to examine subgroups and interactions	3
		(*c*) Explain how missing data were addressed	3
		(*d*) If applicable, explain how loss to follow-up was addressed	
		(*e*) Describe any sensitivity analyses	3
**Results**			
Participants	13*	(a) Report numbers of individuals at each stage of study—eg numbers potentially eligible, examined for eligibility, confirmed eligible, included in the study, completing follow-up, and analysed	4
		(b) Give reasons for non-participation at each stage	4
		(c) Consider use of a flow diagram	Fig. 1
Descriptive data	14*	(a) Give characteristics of study participants (eg demographic, clinical, social) and information on exposures and potential confounders	4

		(b) Indicate number of participants with missing data for each variable of interest	4 /6
		(c) Summarise follow-up time (eg, average and total amount)	4
Outcome data	15*	Report numbers of outcome events or summary measures over time	6/7
Main results	16	(*a*) Give unadjusted estimates and, if applicable, confounder-adjusted estimates and their precision (eg, 95% confidence interval). Make clear which confounders were adjusted for and why they were included	6/7
		(*b*) Report category boundaries when continuous variables were categorized	
		(*c*) If relevant, consider translating estimates of relative risk into absolute risk for a meaningful time period	
Other analyses	17	Report other analyses done—eg analyses of subgroups and interactions, and sensitivity analyses	7
**Discussion**			
Key results	18	Summarise key results with reference to study objectives	7/8
Limitations	19	Discuss limitations of the study, taking into account sources of potential bias or imprecision. Discuss both direction and magnitude of any potential bias	9
Interpretation	20	Give a cautious overall interpretation of results considering objectives, limitations, multiplicity of analyses, results from similar studies, and other relevant evidence	9/10

Generalisability	21	Discuss the generalisability (external validity) of the study results	9/10
**Other information**			
Funding	22	Give the source of funding and the role of the funders for the present study and, if applicable, for the original study on which the present article is based	10

*Give information separately for exposed and unexposed groups.

Note: An Explanation and Elaboration article discusses each checklist item and gives methodological background and published examples of transparent reporting. The STROBE checklist is best used in conjunction with this article (freely available on the Web sites of PLoS Medicine at http://www.plosmedicine.org/, Annals of Internal Medicine at http://www.annals.org/, and Epidemiology at http://www.epidem.com/). Information on the STROBE Initiative is available at http://www.strobe-statement.org.
